# Multi-Omics Analyses Uncover the Mechanism Underlying Polyploidization-Enhanced Steviol Glycosides Biosynthesis in *Stevia rebaudiana*

**DOI:** 10.3390/plants13182542

**Published:** 2024-09-10

**Authors:** Juan Liu, Jiaxue Wang, Mingjia Chen, Wenna Meng, Anping Ding, Miao Chen, Rongping Ding, Mingpu Tan, Zengxu Xiang

**Affiliations:** 1 College of Life Sciences, State Key Laboratory of Crop Genetics & Germplasm Enhancement, Nanjing Agricultural University, Nanjing 210095, China; 2021116018@stu.njau.edu.cn (J.L.); 2023816123@stu.njau.edu.cn (J.W.); mjchen@njau.edu.cn (M.C.); 2022116038@stu.njau.edu.cn (W.M.); 2 College of Horticulture, Nanjing Agricultural University, Nanjing 210095, China; 2021804301@stu.njau.edu.cn (A.D.); cm20909@stu.njau.edu.cn (M.C.); 2022104135@stu.njau.edu.cn (R.D.)

**Keywords:** polyploidization, metabolome, transcriptome, rhizosphere microbes, UDP-glycosyltransferases

## Abstract

*Stevia rebaudiana* (Bertoni) is a valuable sweetener plant whose sweetness primarily derives from steviol glycosides (SGs), especially rebaudioside A (RA). Polyploidization has the potential to enhance the content of active ingredients in medicinal plants, making this strategy a promising avenue for genetic improvement. However, the underlying regulatory mechanisms that contribute to the fluctuating SGs content between autotetraploid and diploid stevia remain unclear. In this study, we employed metabolic analysis to identify 916 differentially accumulated metabolites (DAMs), with the majority, specifically terpenoids, flavonoids, and lipids, exhibiting upregulation due to polyploidization. Notably, the content of stevia’s signature metabolite SGs (including RA, steviolbioside, and rebaudioside C), along with their precursor steviol, increased significantly after polyploidization. Furthermore, a comprehensive analysis of the transcriptome and metabolome revealed that the majority of differentially expressed genes (DEGs) involved in the SG-synthesis pathway (*ent-KAH*, *ent-KS1*, *UGT73E1*, *UGT74G1*, *UGT76G1*, *UGT85C2*, and *UGT91D2*) were upregulated in autotetraploid stevia, and these DEGs exhibited a positive correlation with the polyploidization-enhanced SGs. Additionally, multi-omics network analysis indicated that several transcription factor families (such as five *NACs*, four *WRKYs*, three *MYBs*, eight *bHLHs*, and three *AP2/ERFs*), various transporter genes (four ABC transporters, three triose-phosphate transporters, and two sugar efflux transporters for intercellular exchange), as well as microorganisms (including *Ceratobasidium* and *Flavobacterium*) were positively correlated with the accumulation of RA and steviol. Overall, our results indicate the presence of a regulatory circuit orchestrated by polyploidization, which recruits beneficial rhizosphere microbes and modulates the expression of genes associated with SG biosynthesis, ultimately enhancing the SG content in stevia. This finding will provide new insights for promoting the propagation and industrial development of stevia.

## 1. Introduction

*Stevia rebaudiana* (Bertoni), a perennial herb plant, holds significant sweetening and medicinal properties, making it commercially important [1]. Originating from South America, this herb has been cultivated across diverse regions, including Russia, Indonesia, USA, India, Canada, Brazil, Japan, China, Mexico, Korea, and Pakistan [2,3]. The leaves of stevia are rich in bioactive compounds, sterols, phytochemicals, and natural antioxidants that exhibit antimicrobial and antihypertensive effects [4]. Notably, the leaves contain secondary metabolites, known as steviol glycosides (SGs), which are approximately 250-300 times sweeter than sucrose [5], with implications for the management of diabetes, obesity, and hypertension [6]. 

SGs are specialized diterpenoid compounds, featuring an aglycone core known as steviol, with the principal components being stevioside and rebaudioside A (RA) [7]. RA is especially sought after for its high sweetness and palatable flavor [6,8,9]. The biosynthesis of SGs is a complex process that involves two principal stages: the formation of the backbone structure and its subsequent glycosylation [10]. The diterpenoid aglycone (e.g., steviol) backbone formation is catalyzed by enzymes like *ent*-kaurene synthase (*ent*-KS), *ent*-kaurene oxidase (*ent*-KO), and *ent*-kaurenoic acid hydrolase (*ent*-KAH). Subsequently, glycosylation reactions occur at the steviol positions of the skeleton, which are catalyzed by UDP-glycosyltransferases (UGTs) [1]. The synthesis of the diterpenoid steviol shares a common biosynthetic pathway with other diterpenes originating from geranylgeranyl diphosphate [11]. The cytosolic UGTs (such as *UGT73E1*, *UGT74G1*, *UGT76G1*, *UGT85C2,* and *UGT91D2*) play a critical role in glycosylation reactions that embellish the steviol backbone at specific hydroxyl and carboxylic acid groups, diversifying SGs and their sweetness profiles [4,10].

The rhizospheres, serving as the dynamic interface between plant roots and the soil environment, harbor a remarkably diverse array of functional microorganisms [12,13]. These rhizosphere microbiomes play a pivotal role in maintaining host plant fitness and, intriguingly, plant-recruited rhizobacteria were demonstrated to be associated with stevia biomass and SG content under abiotic stresses [8]. Root exudates, released by plants, mediate key interactions with the microbial community, specifically attracting beneficial microorganisms [14]. These microorganisms, designated as plant growth-promoting microorganisms (PGPMs), are instrumental in supporting various plant functions, such as nutrient solubilization, growth promotion, inhibition of pathogen growth, and activation of the plant’s immune system response [15]. Inoculating stevia plants with rhizosphere-isolated PGPMs (*Bacillus polymixa*, *Pseudomonas putida*, and *Azotobacter chroococcum*) significantly enhanced the levels of stevioside and chlorophyll and the macronutrient content including nitrogen and phosphorus [16]. Similarly, phosphorus-solubilizing bacteria isolated from the rhizosphere of stevia plants were found to enhance plant growth, stevioside, and RA content, as well as phosphorus uptake [17]. Microbes in the rhizosphere confer disease protection, promote growth through phytohormone production, and augment plant resilience against environmental fluctuations [18,19]. For instance, the unique core microbiota associated with wild *Glycyrrhiza uralensis* is conjectured to facilitate the metabolism of its characteristic secondary metabolites [20]. The integrated application of endophytic fungi symbiosis and foliar spermidine sprays has demonstrated positive impacts on the growth of stevia plants, especially in terms of enhancing SG yield [21]. Furthermore, arbuscular mycorrhizal fungi (AMF) symbiosis represents a robust strategy for enhancing crop production and quality through improved nutrient absorption [5]. Specifically, AMF has been shown to promote leaf dry biomass production and increase SG yield [5].

Polyploidization, a pervasive phenomenon among angiosperms, introduces new regulatory pathways via genetic alterations, contributing significantly to plant phenotypic and genetic diversity [22,23]. This process has been recognized as a potent method for amplifying the yield of active ingredients in medicinal plants [24]. Accumulating evidence suggests that polyploid plants frequently showcase superior phenotypic traits compared to their diploid counterparts, such as increased stress tolerance, higher active compound levels, and augmented vigor [25,26,27]. For instance, tetraploid *Isatis indigotica* presents elevated antiviral components (coniferyl alcohol and lariciresinol glucosides) than diploid *I. indigotica* [28]. In *Lonicera japonica*, the phenolic acid, flavonoid content, and antioxidant capacity increased post-polyploidization [24]. Yet, the factors underlying the disparities in RA accumulation between diploid stevia and its autotetraploid descendants remain elusive, as do the interactions among gene expression, microbial communities, and SG metabolite accumulation post-polyploidization.

In this study, we embarked on an exhaustive multi-omics analysis to comprehensively examine the differences between diploid and autotetraploid stevia. Our investigation encompassed a wide-targeted metabolomic approach for evaluating metabolite fluctuations, used RNA sequencing to identify transcriptional alterations, and utilized 16S and ITS-based amplicon sequencing to elucidate variations in the rhizosphere microbiome. Initially, our comparative metabolomic analysis unveiled distinct differences in terpenes, flavonoids, phenolic acids, and alkaloid profiles between diploid and autotetraploid stevia, with the latter exhibiting enhanced SG (RA, steviolbioside, and rebaudioside C) levels, and this was attributed to upregulated genes in SG biosynthetic pathway (including *ent-KAH*, *ent-KS1*, *UGT73E1*, *UGT74G1*, *UGT76G1*, *UGT85C2*, and *UGT91D2*) through combining with transcriptome profiles. Furthermore, our multi-omics regulatory network revealed a comprehensive regulatory model depicting the strong associations between root-associated microbial genera, such as *Ceratobasidium* and *Flavobacterium*, and SG accumulation. This integrated analysis underscores the intricate gene–metabolite–microorganism interplay regulating SG production in stevia post-polyploidization. Overall, our findings provide valuable insights into the regulation of genes, metabolites, and microorganisms in stevia plants of varying ploidy, which can guide the cultivation of high-quality stevia cultivars.

## 2. Results

### 2.1. Differentially Accumulated Metabolites (DAMs) in Stevia Post-Polyploidization

**Figure 1:**
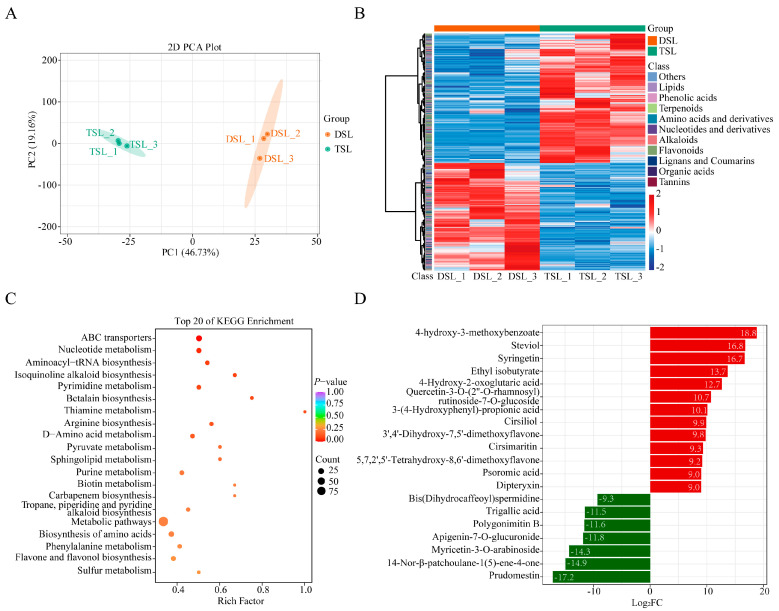
Differentially accumulated metabolites (DAMs) in stevia post-polyploidization. (**A**) PCA score plot. (**B**) Heatmap of DAMs in stevia post-polyploidization. (**C**) KEGG enrichment analysis of DAMs. (**D**) Top 20 altered metabolites in stevia post-polyploidization. Log_2_FC means log_2_FoldChange (TSL/DSL). DSL represents diploid stevia leaf and TSL for autotetraploid stevia leaf.

To investigate the effects of polyploidization on metabolite fluctuations, UPLC-MS/MS-based targeted metabolic profiling was employed for analyzing metabolite content in stevia autotetraploid and its diploid counterparts (Shoutian3, 2n = 22). The results of multivariate principal component analysis (PCA) of the detected metabolites revealed a distinct pattern of metabolite clustering between the diploid and autotetraploid stevia (Figure 1A). A total of 916 metabolites showing significant accumulation differences (|log_2_FC| ≥ 0.6, VIP ≥ 0.9) between diploid and autotetraploid were identified as differentially accumulated metabolites (DAMs), of which 522 were upregulated post-polyploidization (Figure 1B, Table S1). Kyoto Encyclopedia of Genes and Genomes (KEGG) pathway enrichment analysis results revealed significant enrichment of these DAMs in pathways such as amino acids biosynthesis, flavone and flavonol biosynthesis, pyrimidine metabolism, nucleotide metabolism, and ABC transporters (Figure 1C). Polyploidization demonstrably affected the primary and secondary metabolites of stevia leaf, albeit to varying degrees (Table S2). Compared with the diploid parent, a considerable proportion of DAMs in the category of nucleotides and their derivatives (87.5%), flavonoids (62.6%), lipids (66.7%), terpenoids (74.5%), and vitamins (66.7%) were upregulated in autotetraploid. However, 57.8% of phenolic acid and 55.8% of alkaloids exhibited a down-regulation pattern post-polyploidization (Table S2). 

Regarding the polyploidization-sensitive flavonoids, several compounds such as syringetin, quercetin-3-O-rutinoside-7-O-glucoside, 3′,4′-dihydroxy-7,5′-dimethoxyflavone, cirsiliol, cirsimaritin, and 5,7,2′,5′-tetrahydroxy-8,6′-dimethoxyflavone were significantly upregulated in autotetraploid stevia compared to diploid stevia (Table 1). In terms of lipids, both sphingolipids and lysoPE were mostly upregulated post-polyploidization (Table 1). Notably, various vitamin metabolites including vitamin A, nicotinamide, riboflavin, and biotin were markedly upregulated in autotetraploid stevia (Table 1). Furthermore, four DAMs (RA, steviol, steviolbioside, and rebaudioside C) in the SG synthesis pathway, were accumulated in higher amounts in autotetraploid stevia. Of particular interest, steviol, the vital precursor in the SG synthesis pathway, also exhibited a significant increase in autotetraploid stevia and emerged as the second most upregulated metabolite (Figure 1D).

### 2.2. Differentially Expressed Genes (DEGs) in Stevia Post-Polyploidization

**Figure 2:**
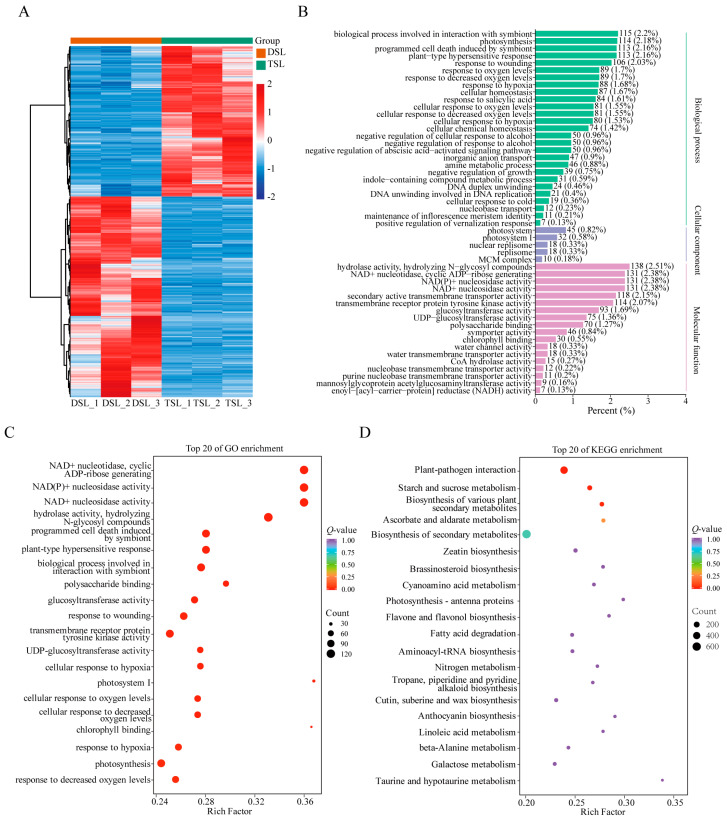
Differentially expressed genes (DEGs) in stevia post-polyploidization. (**A**) Heatmap of DEGs based on hierarchical clustering analysis. (**B**,**C**) GO enrichment analysis of DEGs. (**D**) KEGG enrichment pathway analysis of DEGs. DSL represents diploid stevia leaf and TSL for autotetraploid stevia leaf.

To further elucidate the influence of polyploidization on gene expression and secondary metabolite synthesis, we conducted a comparative transcriptomic analysis of diploid and autotetraploid stevia leaves, and 7153 differentially expressed genes (DEGs) were identified (|Log_2_FC| ≥ 1, *p*-value < 0.05, Figure 2A, Table S3). Furthermore, 11 randomly selected DEGs were verified by qRT-PCR; the results showed that the expression trend of DEGs detected by qRT-PCR was consistent with the transcriptional sequencing data (Figure S1). To identify putative functions and related biological pathways of potential transcripts, we performed Gene ontology (GO) and KEGG enrichment analysis of DEGs. The results of the GO enrichment analysis indicated that DEGs were primarily enriched in “hydrolase activity, hydrolyzing N-glycosyl compounds”, “biological process involved in interaction with symbiont”, “photosynthesis”, “glucosyltransferase activity”, and “UDP-glucosyltransferase” (Figure 2B,C). Further, the results of the KEGG pathway enrichment analysis showed that DEGs were significantly enriched in “the plant-pathogen interaction”, “starch and sucrose metabolism”, and “biosynthesis of various plant secondary metabolites” (Figure 2D). 

To gain insight into the molecular mechanisms underlying the biosynthesis of major secondary metabolites in stevia affected by polyploidization, we concentrated on the expression patterns of *UGTs* involved in flavonoids and terpenoid biosynthesis. Our results showed that 26 *UGTs* exhibited significantly different expression patterns between diploid and autotetraploid stevia (Table S4), among which five *UGTs* (*UGT73E1*, *UGT74G1*, *UGT76G1*, *UGT85C2*, and *UGT91D2*) for SG biosynthesis showed pronounced higher expression in autotetraploid stevia than in diploid stevia (Figure S2). Furthermore, *UGT73E1* (Streb.1G038320 and Streb.1G038330) related to the flavonoid and flavonol biosynthetic pathways, and two *UGTs* (*UGT91A1* and *UGT91D2*) related to the anthocyanin biosynthetic pathway, were also upregulated post-polyploidization in stevia (Figure S2). Overall, the coordinated upregulation of *UGT* genes may actively promote the synthesis and accumulation of terpenoids and flavonoids.

Regarding transcription factors (TFs) and transporters involved in polyploidization responses, a total of 201 TFs and 107 transporter DEGs responding to polyploidization were identified (Tables S5 and S6). In general, the numbers of DEGs encoding WRKY, NAC, MYB, bZIP, bHLH, AUX/IAA, AP2/ERF, HSF, HMG, C2H2, MADS-MIKC, mTERF, and other TFs in diploid and autotetraploid were determined (Table S5). Among them, the majority of DEGs in AP2/ERF, MYB, NAC, bZIP, and bHLH family members were upregulated in autotetraploid stevia, and seven DEGs (such as *NAC83_ARATH*, *MYB111_ARATH*, *MYB4_ORYSJ*, *BZP44_ARATH*, *ILR3_ARATH*, *BIM2_ARATH*, and *AP21_ORYSI*) were all markedly upregulated among the differentially expressed TFs (Table 2). Three CorA-like Mg^2+^ transporter genes (Streb.2G000140, Streb.9G031220, and Streb.11G025820) and three sugar efflux transporter genes for intercellular exchange (Streb.11G000040, Streb.1G032140, and Steb.2G031580) were upregulated in autotetraploid stevia (Table 3). In addition, almost all ABC transporters and all triose-phosphate transporter family’s genes were upregulated in autotetraploid, among which *AB11G_ARATH* (Streb.5G025360), *AB10I_ARATH* (Streb.3G009500), *GFT1_ARATH* (Streb.5G032270), and *UGNT1_ARATH* (Streb.6G014120) were significantly upregulated post-polyploidization (Table 3).

### 2.3. Integrative Transcriptome and Metabolome Analysis Revealed the Enhanced SG Biosynthesis in Stevia Post-Polyploidization

To comprehend the regulatory network governing the polyploidization response in stevia, we endeavored to amalgamate transcriptome and metabolome data. KEGG enrichment pathway analysis showed that DEGs and DAMs were mainly enriched in the starch and sucrose metabolism, zeatin biosynthesis, glycolysis/gluconeogenesis, and diterpenoid biosynthesis pathway (Figure S3). 

**Figure 3:**
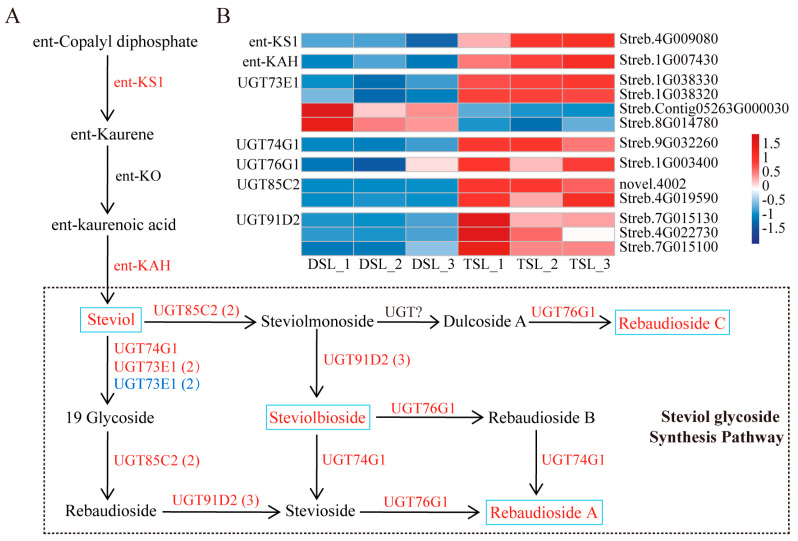
Conjoint transcriptomic and metabolomic analysis of SG biosynthetic pathways in stevia post-polyploidization. (**A**) Diagrammatic representation of genes and metabolites in the SG biosynthetic pathway responsive to polyploidization. Red and blue indicate upregulated and downregulated genes, respectively, and the value in parentheses indicates the number of genes. The polyploidization-upregulated SGs were placed in the lake blue boxes. (**B**) Expression pattern of genes related to SG biosynthetic pathway. DSL represents diploid stevia leaf and TSL for autotetraploid stevia leaf.

When examining the variation in metabolites and genes involved in the SG biosynthesis induced by polyploidization, a coordinate upregulation of four DAMs and 13 DEGs was observed in stevia following polyploidization (Figure 3). Steviol, steviolbioside, RA, and rebaudioside C were markedly altered post-polyploidization (Figure 3A and Table 1). Additionally, several pivotal DEGs encoding *UGTs* (*UGT73E1*, *UGT74G1*, *UGT76G1*, *UGT85C2*, and *UGT91D2*) were considerably affected by polyploidization, and most of them were found to be expressed more in autotetraploid stevia (Figure 3 and Table S7). Interestingly, the expression of *ent-KAH* and *ent-KS1* in the ent-kaurenoic acid biosynthesis pathway upstream of SG synthesis was upregulated post-polyploidization, which explains the higher accumulation of steviol in autotetraploid stevia (Figure 3B). Overall, the conjoint genes–metabolites association analysis revealed a concurrence between transcripts and metabolites, demonstrating that SG biosynthesis was boosted post-polyploidization.

### 2.4. Taxonomic Features of the Rhizosphere Microbes of Diploid and Autotetraploid Stevia

To discern the different rhizosphere bacterial and fungal communities influenced by plant polyploidization, we characterized the taxonomic features of the microbes associated with the rhizosphere of diploid and autotetraploid stevia through 16S rRNA and ITS region sequencing. LEfSe analysis with the threshold of LDA-value > 3.5 and *p*-value < 0.05 was leveraged to pinpoint microbial biomarkers that significantly distinguished the rhizosphere of diploid and autotetraploid stevia. 

**Figure 4:**
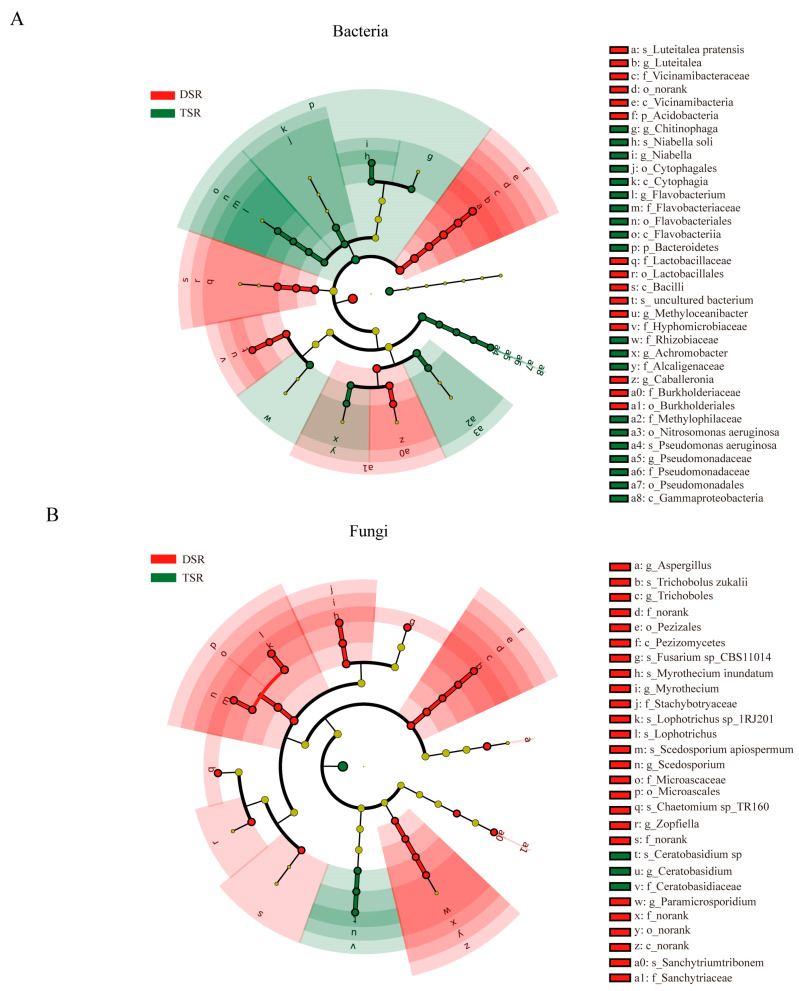
The different enrichment of root-associated microbes between diploid and autotetraploid stevia rhizosphere. (**A**) The different bacterial taxa in diploid and autotetraploid stevia rhizosphere. (**B**) The different fungal taxa in diploid and autotetraploid stevia rhizosphere. The circles from the inner to the outer layers represent the taxonomic level from phylum to species. The dots on the circles represent a term on the corresponding taxonomic level. Significant (red stands for diploid stevia group and green for autotetraploid counterpart) and non-significant (yellow) discriminant taxonomic nodes are colored. Each circle’s diameter reflects the abundance of that taxon in the community. The lowercase p, c, o, f, g, and s in front of the symbol “_” represent the phylum, class, order, family, genus, and species, respectively. DSR, diploid stevia rhizosphere; TSR, autotetraploid stevia rhizosphere.

Consequently, 37 bacterial taxa displayed a significant divergence in abundance between the two groups (Figure 4A, Table S8). At the genus level, *Pseudomonas*, *Niabella*, *Chitinophaga*, *Achromobacter*, and *Flavobacterium* were significantly enriched in autotetraploid stevia rhizosphere (Figure 4A). Delving into the species level, *Pseudomonas aeruginosa* and *Niabella soli* exhibited a pronounced enrichment in autotetraploid stevia rhizosphere, whereas *Luteitalea pratensis* was more prevalent in its diploid counterpart. 

Moving to the fungal realm, the LEfSe analysis revealed that 29 abundant fungal taxa varied substantially in abundance post-polyploidization (Figure 4B, Table S9). Comparative fungal assemblage analysis showed that *Ceratobasidium* was the notably enriched genus in the autotetraploid rhizosphere, whereas *Myrothecium*, *Lophotrichus*, *Myrothecium*, *Scedosporium*, *Fusarium*, *Aspergillus*, and *Chaetomimum* were predominantly associated with diploid equivalence (Figure 4B). In finer detail, one species of *Ceratobasidium* was prevalent in autotetraploid stevia rhizosphere, whereas species including *Chaetomium spTR160*, *Scedosporium apiospermum*, *Lophotrichus sp1RJ2014*, *Myrothecium inundatum*, *Fusarium spCBS110145*, and *Trichobolus zukalii* were characteristic of the diploid stevia rhizosphere (Table S9).

### 2.5. Multi-Omics Network Analysis of the Microbe–Gene–Metabolite Associations

Through a comprehensive integration of transcriptomic, metabolomic, and microbiome analyses, our study has deciphered the complex regulatory network for DAMs, DEGs, and microbial changes influencing SG synthesis in response to stevia polyploidization. By examining gene–metabolite–microbial associations with significant coefficient correlation (|PCC| > 0.80 and *p*-value < 0.05, Table S10), we have elucidated the profound impact of polyploidization on the accumulation of key product RA in the SG metabolism pathway, its precursor steviol, and the intermediate product steviolbioside in the SG synthetic pathway. Autotetraploid stevia rhizosphere exhibited a marked shift in the content of pivotal marker metabolites compared to diploid stevia, and substantially altered expressions were also observed for the essential genes implicated in the SG synthesis pathway. These genetic alterations extended to various TFs and transporters, reflecting a nuanced regulatory adaptation to polyploidization. Moreover, the recruitment of rhizosphere microorganisms displayed striking variations in abundance and diversity between stevia plants of different ploidies. 

**Figure 5:**
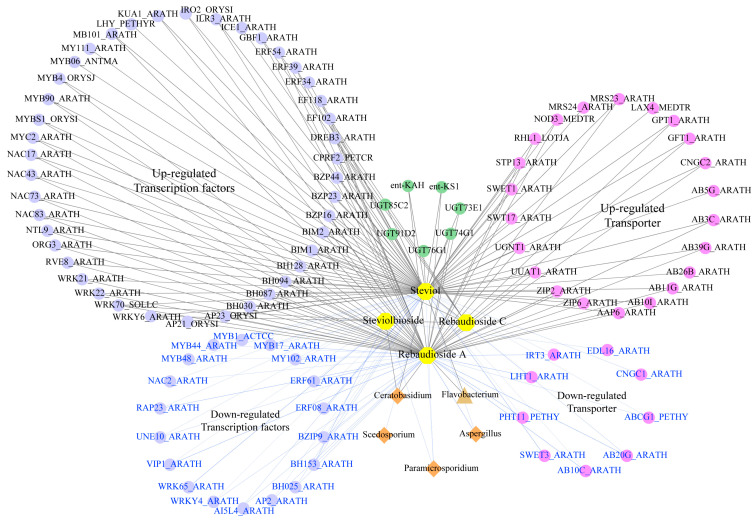
Multi-omics network about the comprehensive gene–metabolite–microorganism associations. Circles represent genes, hexagons represent metabolites, triangles represent bacteria, and diamonds represent fungi. The gray line and blue line represent positive correlation and negative correlation, respectively, and the thickness of the line indicates the strength of the correlation. The thicker the line, the stronger the correlation.

The correlation network analysis shed light on the upregulation of *UGT* genes (*UGT85C2*, *UGT91D2*, *UGT74G1*, *UGT76G1*, and *UGT73E1*), *ent-KS1*, and *ent-KAH*, which exhibited significant positive correlations with the accumulation of SGs (RA, steviolbioside, and rebaudioside C) and their precursor steviol (Figure 5). The analysis also spotlighted several transcription factor families, including NAC, WRKY, MYB, bHLH, and AP2/ERF, which demonstrated substantial positive correlations with SG levels. These polyploidization-upregulated TFs included five NAC family members (*NAC83_ARATH*, *NTL9_ARATH*, *NAC17_ARATH*, *NAC43_ARATH*, and *NAC73_ARATH*), four WRKY family members (*WRKY6_ARATH*, *WRKY21_ARATH*, *WRKY22_ARATH*, and *WRKY70_SOLLC*), three MYB family members (*MY111_ARATH*, *MYB4_ORYSJ*, and *KUA1_ARTAH*), eight bHLH family members (*ORG3_ARATH*, *RHL1_ARATH*, *MYC2_ARATH*, *BIM2_ARATH*, *BIM1_ARATH*, *ILR3_ARATH*, *BH087_ARATH*, and *BH094_ARATH*), and three AP2/ERF family members (*AP23_ORYSI*, *EF118_ARATH*, and *DREB3_ARATH*) (Figure 5). In addition, the interaction network analysis revealed that several transporters were also significantly positively correlated with SG accumulation. Specifically, the interaction network highlighted a significant positive correlation with SGs for four ABC transporters genes (*AAP6_ARATH*, *AB10I_ARATH*, *AB11G_ARATH*, *AB39G_ARATH*, and *AB3C_ARATH*), three triose-phosphate transporter genes (*GPT1_ARATH*, *GFT1_ARATH*, and *UGNT1_ARATH*), and two sugar efflux transporters genes for intercellular exchange (*NOD_MEDTR* and *SWET1_ARATH*) (Figure 5). Conversely, within the negative correlation network, certain downregulated TFs and transporters were identified as potential negative regulators of SG accumulation. From a rhizosphere microorganisms’ perspective, bacteria such as *Flavobacterium* and fungi including *Ceratobasidium* were positively correlated with SG metabolites, and these microorganisms exhibited a pronounces enrichment in the autotetraploid rhizosphere (Figure 5, Tables S8 and S9).

## 3. Discussion

### 3.1. Steviol Glycosides Biosynthesis Mechanism Elucidated through Integrated Transcriptome and Metabolome Analysis

In the wake of plant polyploidization, a spectrum of phenotypic variations frequently emerges, regulated by diverse genetic mechanisms that contribute to the successful natural occurrence or targeted utilization of polyploids [29]. Polyploidization often leads to distinct patterns of primary or secondary metabolite accumulation [30]. In this study, a substantial increase in the accumulation of flavonoids, lipids, and terpenoids occurred in stevia post-polyploidization (Figure 1B, Table S1). This observation aligns with previous research indicating that polyploidization tends to enhance the accumulation of characteristic metabolites in medicinal plants, including *Isatis indigotica* [28], *Lonicera japonica* [24], and *Rheum officinale* [22]. In particular, our results confirmed that autotetraploid stevia exhibited significantly higher SGs, including RA, steviolbioside, and rebaudioside C, with their precursor steviol being notably upregulated in response to polyploidization (Figure 1D, Table 1). Considering the increasing importance of stevia as a potential source of non-caloric natural sweeteners [31], the higher levels of SGs found in autotetraploid stevia offer great potential for broader utilization in the natural food and beverages industry. 

While the genesis of novel polyploidy variants is not fully comprehended, gene expression changes are believed to play a pivotal role [22,30]. There is increasing evidence that alterations in the expression of *UGTs* correlate with variations in SG content and profile [11,32,33]. For example, the upregulation of *SrUGT74G1* by chitosan treatment led to increased production of stevioside and rebaudioside C [32]. Moreover, a positive correlation was observed between the concentrations of rebaudioside A, steviol, and stevioside, particularly upon the co-overexpression of *SrUGT76G1* and *SrKO* genes [11]. Additionally, alginate oligosaccharides promoted SG biosynthesis by up-regulating *UGT74G1*, *UGT85C2,* and *UGT91D2* expression [33]. In this study, we identified five polyploidization-induced *UGTs* (*UGT73E1*, *UGT85C2*, UGT91D2, *UGT74G1*, and *UGT76G1*) (Figure 3) involved in the synthesis of diverse SGs [4,10]. Furthermore, a significant positive correlation was observed between the expression levels of these five *UGTs* and SG contents (RA, steviolbioside, and rebaudioside C), as well as steviol (Figure 5, Table S10). Alongside the five *UGTs* aforementioned, polyploidization induced the expression of *ent-KS* and *ent-KAH*, which have been previously reported to play a role in RA formation (Figure 3) [10]. Therefore, polyploidization appears to be an intriguing approach for enhancing the accumulation of these valuable compounds by improving the expression of corresponding biosynthetic genes.

SGs are diterpenoid compounds, and, with the elucidation of terpenoid synthesis pathways, specific TFs, like WRKY, MYB, bHLH, AP2/ERF, and NAC, which regulate these terpenoid synthesis routes have gained attention in plants [34,35]. In stevia, five *SrbHLH* TFs (*SrbHLH22*, *SrbHLH111*, *SrbHLH126*, *SrbHLH142*, and *SrbHLH152*) have been identified as activators of *UGT76G1* expression, while *SrbHLH6*, *12*, *60*, *72*, *83*, *85*, *134*, *147*, and *148* are implicated in regulating the expression of other structural genes central to RA biosynthesis [6]. Given that TFs serve as crucial regulatory factors in terpenoid biosynthesis, variations in their expression levels between autotetraploid and diploid stevia may influence diterpene SGs. To explore additional regulatory factors that may impact SG biosynthesis post-polyploidization, we constructed a correlation network that ties together SGs and TFs within a key network based on genes and metabolites. In autotetraploid stevia, several differentially expressed TFs, including members of the NAC, WRKY, MYB, bHLH, and AP2/ERF families, were identified as potential regulators in promoting RA synthesis (Figure 5). Importantly, the majority of the TFs activated by polyploidization exhibited a positive correlation with augmented SG production observed in autotetraploid stevia (Figure 5). These data were reminiscent of the role of *SrbHLHs* in transcriptionally activated structural genes involved in RA biosynthesis [6]. Such results suggested that the TFs aforementioned as key regulatory factors participate in SG biosynthesis. However, further research is necessary to ascertain whether these TFs directly regulate *SrUGT76G1* and the subsequent SG accumulation. Investigating the regulatory mechanisms of these TFs through overexpression or knockout studies could provide critical insights into how polyploidization enhances SG synthesis.

### 3.2. Optimized Rhizosphere Microbiome Contributes to SG Biosynthesis in Autotetraploid Stevia

Recently, the study of microbial community structure and functional capabilities associated with the sustainable cultivation of economically important medicinal plants has attracted considerable attention. For example, endophytes have been found to boost the growth of *Anoectochilus roxburghii* and elevate the levels of its active ingredients [36]. *Bacillus* strains, as endophytes, have been found to improve the vegetative growth of *Gloriosa superba* and raise the production of bioactive secondary metabolites like colchicine and gloriosine [37]. Stevia plants treated with a combination of *Azospirillum brasilense* and *Bacillus cereus* exhibited increased plant height, leaf number, antioxidant activity, carbohydrates, protein levels, and the expression of SG genes [38]. Intriguingly, the phenotypic changes induced by polyploidization may lead to the formation of novel microbial combinations [39]. While studies have examined the effects of polyploidization on individual root bacteria and fungi, the overall microbial community of rhizospheres remains under-characterized. Research on neopolyploids has highlighted a greater abundance of key bacterial taxa in their microbiomes compared to diploids [40]. Notably, we found that polyploidization leads to an increase in the abundance and diversity of stevia rhizospheric bacteria, with the genera *Pseudomonas*, *Niabella*, *Chitinophaga*, *Achromobacter*, and *Flavobacterium* being significantly enriched in autotetraploid rhizospheres (Figure 4A and Table S8). *Flavobacterium*, in particular, demonstrates a notable capacity for transforming rhizosphere-associated phosphorus through specialized uptake and degradation systems [41]. Terrestrial *Flavobacterium* strains have evolved mechanisms to interact with plant roots beneficially, contributing to plant health [42]. Microbial communities significantly influence plant performance and range expansion [43]. Therefore, understanding how polyploidization alters these communities can offer valuable insight into the mechanisms driving the SG accumulation in stevia.

To delve deeper into the influence of microbial diversity on SG biosynthesis, we constructed a correlation network between SGs and microorganisms. Our findings indicated a strong positive correlation between the abundance of *Flavobacterium* in the bacterial community, as well as *Ceratobasidium* and *Gigaspora* in the fungal community, with RA levels (Figure 5). We also identified several marker microorganisms associated with the accumulation of RA in autotetraploid stevia (Figure 5), including *Pseudomonas aeruginosa* [44] and *Ceratobasidium* [45], which have been reported to be PGPMs. *Ceratobasidium sp. AR2*, for instance, has been demonstrated to enhance the content of bioactive components in *Anoectochilus roxburghii* [46]. Additionally, KEGG and GO enrichment analysis revealed that most DEGs were significantly enriched in pathways related to the plant–pathogen interaction and biological process involved in interaction with symbiont pathways (Figure 2B,D), indicating that microorganisms may perturb genetic changes and jointly contribute to the accumulation of SGs. Therefore, future research will focus on isolating these beneficial microbes from autotetraploid stevia rhizosphere and verifying their role in the expression of key *UGTs* genes and the accumulation of active ingredients in SG synthesis by inoculating the separated microorganisms into the roots of stevia. Moreover, the isolation of these valuable microorganisms and the development of associated microbial fertilizers or regulators should be performed in future studies to artificially alter rhizosphere microecology and enhance SG productivity.

## 4. Materials and Methods

### 4.1. Plant Materials

The experimental materials for this study consisted of diploid (Shoutian3, 2n = 22) and autotetraploid (4n = 44) varieties of *Stevia rebaudiana*. Autotetraploid stevia was specifically created in the laboratory through the induction of diploid stevia (Shoutian3) production using colchicine [47]. These plants were transplanted from tissue culture seedlings to the experimental field of the Traditional Chinese Medicine Biotechnology Laboratory of Nanjing Agricultural University, where they were cultivated under natural light conditions for approximately one year. Given that the leaves constitute the primary active component of stevia, we focus our collection efforts on leaves from both diploid and autotetraploid stevia plants. The third and fourth leaves, from top to bottom, were sampled from the same position of diploid and autotetraploid stevia, and three biological replicates were sampled, immediately snap-frozen in liquid nitrogen, and stored at −80 °C for subsequent metabolomics and transcriptomic analysis. Additionally, we simultaneously collected stevia rhizosphere soil samples from different ploidy levels. These samples were placed in 5 mL sterile centrifuge tubes containing sterile water, and the rhizosphere microorganisms were collected for 16S and ITS sequencing following centrifugation (2500× *g*, 10 min), with three biological replicates for each ploidy type.

### 4.2. Total RNA Extraction, Library Preparation, and Transcriptome Sequencing

Total RNA was extracted from young leaf samples (80 mg each) of two different stevia (diploid and autotetraploid) using the CTAB-PBIOZOL reagent, according to the manual instructions. A Nano Drop and Agilent 2100 bioanalyzer (Thermo Fisher Scientific, Waltham, MA, USA) was used to check and measure the purity and concentration of the isolated RNA. Then, total RNA integrity was assessed using the RNA Nano 6000 Assay Kit of the Bioanalyzer2100 system (Agilent Technologies, Santa Clara, CA, USA). The mRNA was purified using Oligo (dT)-attached magnetic beads and enriched with PCR to prepare the sequencing library. The PCR products were purified (AMPure XP system, Beckman Coulter Life Sciences, Indianapolis, IN, USA) and library quality was assessed on the Agilent Bioanalyzer 2100 system. The cDNA library was then subjected to deep RNA sequencing (RNA-seq) on the Nova Seq 6000 Illumina HiSeq Platform (Illumina, San Diego, CA, USA), generating paired-end reads of 150 bp.

### 4.3. Transcriptome Assembly, Differential Expression, and Functional Enrichment Analysis

The raw RNA reads containing adapters, ambiguous nucleotides (N), and low-quality bases were removed to obtain the high-quality clean reads using fastp 0.21.0 [48]. The clean reads were aligned to the stevia genome [10] using HISAT2 [49] with default parameters. Gene expression levels were assessed by determining the fragments per kilobase of transcript per million mapped reads (FPKM) for each gene, based on the length of each gene [50]. The mapped reads of each sample were assembled and quantified using StringTie tools [51]. 

Differentially expressed genes (DEGs) were analyzed by DESeq2 software (V 1.44.0) [52], and the Benjamini and Hochberg method was used to adjust the *p*-value [50]. The |log2FC| ≥ 1 and adjusted *p*-value < 0.05 were used as the threshold for identifying DEGs. The functional-enrichment analysis of differentially expressed genes (DEGs) was performed based on the hypergeometric test using the Kyoto Encyclopedia of Genes and Genomes (KEGG; http://www.kegg.jp/kegg, 29 May 2024) and Gene Ontology (GO; https://www.geneontology.org, 29 May 2024) databases [53].

### 4.4. qRT-PCR Analysis

The expression levels of the transcripts were then detected by qRT-PCR. Briefly, the total RNA was extracted and reverse-transcribed into the first-strand cDNA using the Hiscript^®^II Q Select RT SuperMix for qPCR Reverse Transcription Kit (R23301, Vazyme, Nanjing, JS, China). Quantitative real-time PCR (qPCR) was performed using synthetic cDNA as a template and mRNA expression was normalized to internal reference gene Actin with the 2^−ΔΔCT^ method [54]. The primers used for qPCR analysis are listed in Table S11.

### 4.5. Metabolite Measurements and Analysis

The UPLC-ESI-MS/MS system (UPLC, ExionLC™ AD, Framingham, MA, USA) and Tandem mass spectrometry system (MS, Applied Biosystems 6500 QTRAP, Foster, CA, USA) were used to carry out the metabolite determination with Agilent SB-C18 column (1.8 µm, 2.1 mm × 100 mm) [55]. The mobile phase consisted of solvent A, pure water with 0.1% formic acid, and solvent B, acetonitrile with 0.1% formic acid. The gradient elution was as follows: 0–10 min, 5% A and 95% B; 10–11.1 min, 5–95% A and 95–5% B; 11.1–14 min, 95% A and 5% B. The flow velocity was set at 0.35 mL per minute, the column oven temperature was set to 40 °C, and the injection volume was 2 µL. 

The effluent was connected to an ESI-triple quadrupole-linear ion trap (QTRAP)-MS. The ESI source was operated with the following parameters: source temperature 500 °C; ion spray voltage (IS) 5500 V (positive ion mode)/−4500 V (negative ion mode); gas I, gas II, and curtain gas were set at 50, 60, and 25 psi, respectively; the collision-activated dissociation was high. Triple quadrupole scans were acquired using predefined multi-reaction monitoring (MRM) experiments with collision gas (nitrogen). The declustering potential and collision energy for each individual MRM transition were further optimized. A specific set of MRM transitions was monitored for each period based on the metabolites eluted within that period. 

Metabolite mass spectrometry data were collected for diploid and autotetraploid stevia samples. The relative abundance of metabolites is calculated by integrating and correcting the mass spectral peaks corresponding to the same metabolite in different samples. Principal component analysis (PCA) in each comparison group was performed by R package [50]. To analyze the diploid and autotetraploid groups, differentially accumulated metabolites (DAMs) were identified by |log_2_FC| ≥ 0.6 and variable importance plot (VIP) ≥ 0.9. The functional pathway enrichment analysis of these DAMs was conducted using the KEGG database [53].

### 4.6. Rhizosphere Microbial DNA Extraction and Amplicon Sequencing

To isolate rhizosphere microbial communities, the rhizoplane samples were first soaked with ddH_2_O and then centrifuged to collect all of the sediment from the root surfaces, which were treated as rhizosphere microbial samples. The microbial DNA of the rhizosphere was extracted from diploid and autotetraploid stevia using the E.Z.N.A.^®^ Soil DNA Kit (Omega Bio-tek, Norcross, GA, USA) according to the manufacturer’s protocols. DNA quality and quantity were determined using a NanoDrop 2000 Spectrophotometer (Bio-Rad Laboratories Inc., Hercules, CA, USA) [56]. The 16S rRNA gene was amplified for each sample in a PCR using the genomic DNA as a template and bacterial universal primers 27F 5′-AGRGTTYGATYMTGGCTCAG-3′ and 1492R 5′-RGYTACCTTGTTACGACTT-3′ [57,58]. The ITS1F (5′-TCCGTAGGTGAACCTGCGG-3′) and ITS4R (5′-TCCTCCGCTTATTGATATGC-3′) primer pair was used to amplify the fungal internal transcribed spacer (ITS) gene [59]. Amplicons were extracted from 2% agarose gels and purified using the AxyPrep DNA Gel Extraction Kit (Axygen Biosciences, Union City, CA, USA), according to the manufacturer’s instructions, and quantified using QuantiFluor™-ST (Promega, Madison, WI, USA). All high-throughput sequencing analyses of bacterial and fungal genes were performed based on the Illumina MiSeq platform (2 × 250 paired ends) at Shanghai Biozeron Biotechnology Co., Ltd. (Shanghai, China).

### 4.7. Microbial Data Analysis

Mothur (version v.1.39.5) was used for quality control and QIIME (V1.9.1) was used for taxonomical analysis to obtain high-quality microbial data [60]. The reads were trimmed by discarding quality scores below 20 and sequence lengths below 400 bp. The unique sequences among these remaining reads were used to define operational taxonomic units (OTUs) using USEARCH (version 7.1) with a threshold of 97% similarity [61]. Linear discriminate analysis (LDA) effect size (LEfSe), performed using the Galaxyonline analytics platform1, was used to analyze the differences in the microbial communities between the diploid and autotetraploid groups in stevia. The significant taxonomical biomarkers were selected with a *p*-value < 0.05 and logarithmic LDA-value > 3.5. A Kruskal–Wallis sum-rank test was performed to examine the changes and dissimilarities among classes followed by LDA analysis to determine the size effect of each distinctively abundant taxa [36].

### 4.8. Multi-Omics Integrative Analysis

KEGG pathway analysis was performed to identify the common metabolic pathways between the DEGs and DAMs. For the integrative multi-omics analysis, Pearson correlation coefficients (PCCs) between DEGs and DAMs, as well as microorganisms, were calculated to determine the correlation between them. A threshold of |PCC| > 0.8 was set to infer meaningful relationships. Important metabolites, critical genes, and dominant microorganisms were selected to construct the gene–metabolite–microorganism correlation network. A network was constructed and visualized with default parameters and similarity thresholds using Cytoscape v3.7.1.

## 5. Conclusions

This study presents a comprehensive analysis of the regulatory networks involved in the biosynthesis of SGs in autotetraploid and diploid stevia plants. Through an exploration of metabolome, transcriptome, and microbiome levels, significant differences between the two stevia varieties were revealed. Notably, autotetraploid stevia was found to accumulate significantly higher levels of SGs (RA, steviolbioside and rebaudioside C) and their precursor steviol compared to diploid stevia. This study also advanced our understanding of the SG biosynthesis pathway and identified a broad stimulation of metabolism following polyploidization. This stimulation resulted in enhancements in SG biosynthesis and provided insights into the complex relationships between responsive genes and metabolic reactions. Furthermore, the multi-omics network analysis highlighted the intricate gene–metabolite–microbe associations regulating SG production in stevia and underscored the critical role of the rhizosphere microbiome in this context. Understanding these complex interactions opens avenues for enhancing SG yields through targeted manipulation of rhizosphere dynamics and genetic pathways in stevia. Overall, this study lays the groundwork for further research on biotechnological approaches for the mass propagation of stevia and its applications in medicine, pharmacy, and the food industry.

## Figures and Tables

**Table 1:** The upregulated differentially accumulated metabolites (DAMs) in autotetraploid stevia compared to diploid stevia.

Compounds	Log_2_FC	VIP
**Terpenoids**		
3β,21β,24-trihydroxyserrat-14-en-29-oic acid-3β(4-hydroxy-3-methoxybenzoate)	18.8	1.5
Steviol	16.8	1.5
Ent-kaurenal	1.9	1.3
Rebaudioside A	0.9	1.4
Steviolbioside	0.8	1.0
Rebaudioside C	0.6	0.9
**Phenolic acids**		
3-(4-hydroxyphenyl)-propionic acid	10.1	1.5
(S)-2-phenyloxirane	2.4	1.4
1-O-feruloyl-3-O-p-coumaroylglycerol	3.1	1.3
5-O-caffeoylshikimic acid	2.1	1.4
Galloyl methyl gallate	2.0	1.2
3-O-feruloylquinic acid	1.82	1.37
**Lipids**		
9,10-dihydroxy-12,13-epoxyoctadecanoic acid	4.4	1.4
Dihydrosphingosine-1-phosphate	3.4	1.3
3-dehydrosphinganine	2.3	1.3
Phytosphingosine	2.1	1.3
LysoPC 18:1	1.6	1.3
LysoPE 18:0	1.7	1.2
1-(9Z-octadecenoyl)-sn-glycero-3-phosphocholine	1.7	1.2
LysoPE 16:1	1.6	1.1
LysoPE 16:1	2.0	1.1
**Flavonoids**		
Syringetin	16.7	1.5
Quercetin-3-O-(2″-O-rhamnosyl) rutinoside-7-O-glucoside	10.7	1.4
Cirsiliol	9.9	1.4
Cirsimaritin	9.3	1.5
3′,4′-dihydroxy-7,5′-dimethoxyflavone	9.8	1.4
Quercetin-3,3′-dimethyl ether	8.1	1.4
5,7,2′,5′-tetrahydroxy-8,6′-dimethoxyflavone	9.2	1.4
3,7-dimethylquercetagetin-(3′,4′,5,6-Tetrahydroxy-3,7-dimethoxyflavone)	8.8	1.4
Rhamnetin; 3,5,3′,4′-tetrahydroxy-7-methoxyflavone	8.0	1.5
**Alkaloids**		
Tryptamine	4.9	1.4
N-caffeoylputrescine	3.6	1.4
Retronecine	2.7	1.4
2-phenylethylamine	2.7	1.4
**Vitamin**		
Retinol	1.4	1.4
Nicotinamide	1.0	1.4
Riboflavin	2.0	1.4
Biotin	2.2	1.4
**Others**		
2-phenylethanol	2.2	1.4
Dipteryxin	9.01	1.45
2-Dehydro-3-deoxy-L-arabinonate	2.14	1.45
D-Threose	2.42	1.43

Log_2_FC means log_2_FoldChange (TSL/DSL); VIP (Variable Importance in Projection) is an index used to evaluate the correlation between a certain metabolite and sample classification. DSL represents diploid stevia leaf and TSL for autotetraploid stevia leaf.

**Table 2:** The differentially expressed TFs post-polyploidization.

ID	Name	Log_2_FC	ID	Name	Log_2_FC
**WRKY**			Streb.2G002460	*BZP23_ARATH*	1.9
Streb.8G004280	*WRK21_ARATH*	2.0	Streb.4G010840	*CPRF2_PETCR*	1.3
Streb.11G007600	*WRK22_ARATH*	1.8	Streb.7G020660	*GBF1_ARATH*	1.2
Streb.1G002040	*WRK70_SOLLC*	1.8	Streb.3G007530	*BZIP9_ARATH*	−3.1
Streb.3G012150	*WRKY6_ARATH*	1.6	Streb.3G012510	*AI5L4_ARATH*	−2.1
Streb.4G015740	*WRKY4_ARATH*	−2.5	Streb.8G015740	*VIP1_ARATH*	−1.0
Streb.2G030570	*WRK51_ARATH*	−1.4	**bHLH**		
Streb.4G005140	*WRK65_ARATH*	−1.4	Streb.Contig05035G000020	*ILR3_ARATH*	6.1
Streb.2G051870	*WRK40_ARATH*	−1.3	Streb.2G020970	*BIM2_ARATH*	4.3
**NAC**			Streb.1G023980	*IRO2_ORYSI*	3.9
Streb.8G026410	*NAC83_ARATH*	9.2	Streb.1G024080	*ORG3_ARATH*	3.8
Streb.10G007870	*NTL9_ARATH*	1.9	Streb.4G026850	*BH087_ARATH*	3.8
Streb.5G033120	*NAC75_ARATH*	1.3	Streb.10G018330	*BH128_ARATH*	3.2
Streb.9G020010	*NAC73_ARATH*	1.3	Streb.2G013370	*BH094_ARATH*	2.4
Streb.8G033830	*NAC17_ARATH*	1.3	Streb.2G028430	*RHL1_LOTJA*	1.4
Streb.10G019010	*NAC43_ARATH*	1.2	Streb.9G004040	*MYC2_ARATH*	1.3
Streb.2G020770	*NAC2_ARATH*	−3.0	Streb.2G055730	*BIM1_ARATH*	1.3
Streb.5G025450	*NAC6_SOYBN*	−1.6	Streb.11G016670	*ICE1_ARATH*	1.3
Streb.7G009790	*NAC91_ARATH*	−1.2	Streb.11G003730	*BH030_ARATH*	1.1
**MYB**			Streb.6G032180	*UNE10_ARATH*	−2.5
Streb.10G031370	*MY111_ARATH*	8.1	Streb.7G020980	*BH025_ARATH*	−1.9
Streb.11G027310	*MYB4_ORYSJ*	4.0	Streb.3G020800	*BH153_ARATH*	−1.7
Streb.11G030580	*KUA1_ARATH*	2.0	Streb.4G022320	*PIF7_ARATH*	−1.5
Streb.6G002150	*MB101_ARATH*	1.9	**AP2/ERF**		
Streb.7G028220	*RVE8_ARATH*	1.8	Streb.5G008600	*AP21_ORYSI*	4.2
Streb.11G013850	*MYB90_ARATH*	1.7	Streb.8G009050	*AP23_ORYSI*	3.3
Streb.2G049910	*MYB06_ANTMA*	1.3	Streb.11G011700	*ERF34_ARATH*	1.7
Streb.4G021750	*MYBS1_ORYSI*	1.1	Streb.4G000620	*ERF54_ARATH*	1.5
Streb.2G045880	*LHY_PETHY R*	1.1	Streb.5G004440	*EF118_ARATH*	1.5
Streb.1G015820	*MYB44_ARATH*	−1.9	Streb.1G037650	*DREB3_ARATH*	1.4
Streb.9G001240	*MYB38_MAIZE*	−1.9	Streb.9G000120	*ERF39_ARATH*	1.1
Streb.2G021360	*MYB1_ACTCC*	−1.9	Streb.10G004210	*EF102_ARATH*	1.1
Streb.9G010620	*MYB48_ARATH*	−1.6	Streb.Contig03456G000010	*ERF61_ARATH*	−2.1
Streb.9G019430	*MY102_ARATH*	−1.2	Streb.10G009110	*RAP23_ARATH*	−1.5
Streb.1G000660	*MYB17_ARATH*	−1.0	Streb.4G017620	*ERF08_ARATH*	−1.5
**bZIP**			Streb.4G017600	*AP2_ARATH*	−1.2
Streb.9G027550	*BZP44_ARATH*	6.1	Streb.6G008530	*ERF3_TOBAC*	−1.0
Streb.10G008960	*BZP16_ARATH*	2.8			

Log_2_FC means log_2_FoldChange (TSL/DSL); DSL represents diploid stevia leaf and TSL for autotetraploid stevia leaf.

**Table 3:** The differentially expressed transporter genes post-polyploidization.

ID	Name	log_2_FC	ID	Name	log_2_FC
**Ion transport protein**			**ABC transporter**		
Streb.3G013310	*CNGC2_ARATH*	3.1	Streb.1G035320	*AB39G_ARATH*	4.2
Streb.2G032360	*CNGC2_ARATH*	1.2	Streb.3G009500	*AB10I_ARATH*	3
Streb.7G022990	*CNGC1_ARATH*	−1.2	Streb.5G025360	*AB11G_ARATH*	2.4
**Sugar (and other) transporter**			Streb.5G001490	*AB3C_ARATH*	1.8
Streb.9G006200	*EDL16_ARATH*	2.1	Streb.4G013640	*AB26B_ARATH*	1.4
Streb.9G025500	*STP13_ARATH*	1.0	Streb.1G016980	*AB5G_ARATH*	1.2
Streb.10G002460	*PHT11_PETHY*	−2.3	Streb.3G008960	*AB3C_ARATH*	1.0
Streb.2G008870	*EDL16_ARATH*	−1.6	Streb.9G030840	*ABCG1_PETHY*	−2.2
**ZIP Zinc transporter**			Streb.10G006460	*AB10C_ARATH*	−1.7
Streb.Contig03477G000010	*ZIP6_ARATH*	5.6	Streb.10G027430	*AB20G_ARATH*	−1.3
Streb.6G017260	*ZIP2_ARATH*	5.7	**Triose-phosphate Transporter**		
Streb.1G047870	*ZIP6_ARATH*	−2.6	Streb.6G014120	*UGNT1_ARATH*	2.5
Streb.10G018100	*IRT3_ARATH*	−1.7	Streb.5G032270	*GFT1_ARATH*	2.3
**CorA-like Mg2+ transporter protein**			Streb.5G022800	*GPT1_ARATH*	1.6
Streb.2G000140	*MRS23_ARATH*	6.6	Streb.9G009400	*UUAT1_ARATH*	1.2
Streb.9G031220	*MRS23_ARATH*	3.6	**Sugar efflux transporter for intercellular exchange**		
Streb.11G025820	*MRS24_ARATH*	1.3	Streb.11G000040	*NOD3_MEDTR*	1.4
**Transmembrane amino acid transporter protein**			Streb.1G032140	*SWET1_ARATH*	1.4
Streb.2G034940	*AAP6_ARATH*	2.3	Streb.2G031580	*SWT17_ARATH*	1.1
Streb.9G013250	*LAX4_MEDTR*	1.1	Streb.8G017430	*SWET3_ARATH*	−1.1
Streb.2G018370	*LHT1_ARATH*	−1.9			

Log_2_FC means log_2_FoldChange (TSL/DSL); DSL represents diploid stevia leaf and TSL for autotetraploid stevia leaf.

## Data Availability

The raw data of RNA-seq, 16S-seq, and ITS-seq that support the conclusions of this article are deposited in NCBI’s Sequence Read Archive (SRA) database. The accession numbers for the datasets are PRJNA1065018 for RNA-seq, PRJNA1064472 for 16S sequencing, and PRJNA1064669 for ITS sequencing. The data will be made publicly available upon publication of this paper.

## Supplementary Materials

The following supporting information can be downloaded at: https://www.mdpi.com/article/10.3390/plants13182542/s1, Figure S1: Verification of gene expression analysis by quantitative real-time PCR (qRT-PCR). *Ent-KAH*: Streb.1G007430; *ent-KS1*: Streb.6G002670; *UGT73E1*(1): Streb.1G038330; *UGT73E1*(2): Streb.Contig05263G000030; *UGT74G1*: Streb.9G032260; *UGT76G1*: Streb.1G003400; *UGT85C2*: Streb.4G019590; *UGT91D2*: Streb.7G015130; *ARF2B_SOLLC*: Streb.2G023640; *DECR2_ARATH*: Streb.1G034220; *XTH23_ARATH*: Streb.6G015550. DSL represents diploid stevia leaf and TSL for autotetraploid stevia leaf; Figure S2: The expression pattern of genes encoding UDP-dependent glycosyltransferase (UGT) in diploid and autotetraploid stevia. The heatmaps was sacked in row direction based on FPKM of genes. DSL represents diploid stevia leaf and TSL for autotetraploid stevia leaf; Figure S3: KEGG pathway enrichment of differentially expressed genes (DEGs) and differentially accumulated metabolites (DAMs) in stevia post-polyploidization; Table S1: Differential accumulated metabolites (DAMs) detected between diploid and autotetraploid of stevia; Table S2: Number of differentially accumulated metabolites (DAMs) in autotetraploid stevia with respect to the diploid parent; Table S3: Differential expressed genes (DEGs) detected between diploid and autotetraploid of stevia; Table S4: Differential expressed genes encoding UDP-dependent glycosyltransferase (UGT); Table S5: Differentially expressed transcription factors in response to polyploidization; Table S6: Differentially expressed transporters in response to polyploidization; Table S7: Differentially expressed genes (DEGs) related to steviol glycosides (SGs) biosynthesis; Table S8: The different bacterial taxa in diploid and autotetraploid stevia rhizosphere; Table S9: The different fungal taxa in diploid and autotetraploid stevia rhizosphere; Table S10: The correlated network of genes and microorganisms with steviol glycosides (SGs) in stevia post-polyploidization; Table S11: Primers used for qRT-PCR.
