# Supplementary figures and images for: Multi-Omics Analyses Uncover the Mechanism Underlying Polyploidization-Enhanced Steviol Glycosides Biosynthesis in Stevia rebaudiana
Source: Plants (Basel). 2024 Sep 10;13(18):2542. doi: 10.3390/plants13182542 (PMC11434884; doi:10.3390/plants13182542)

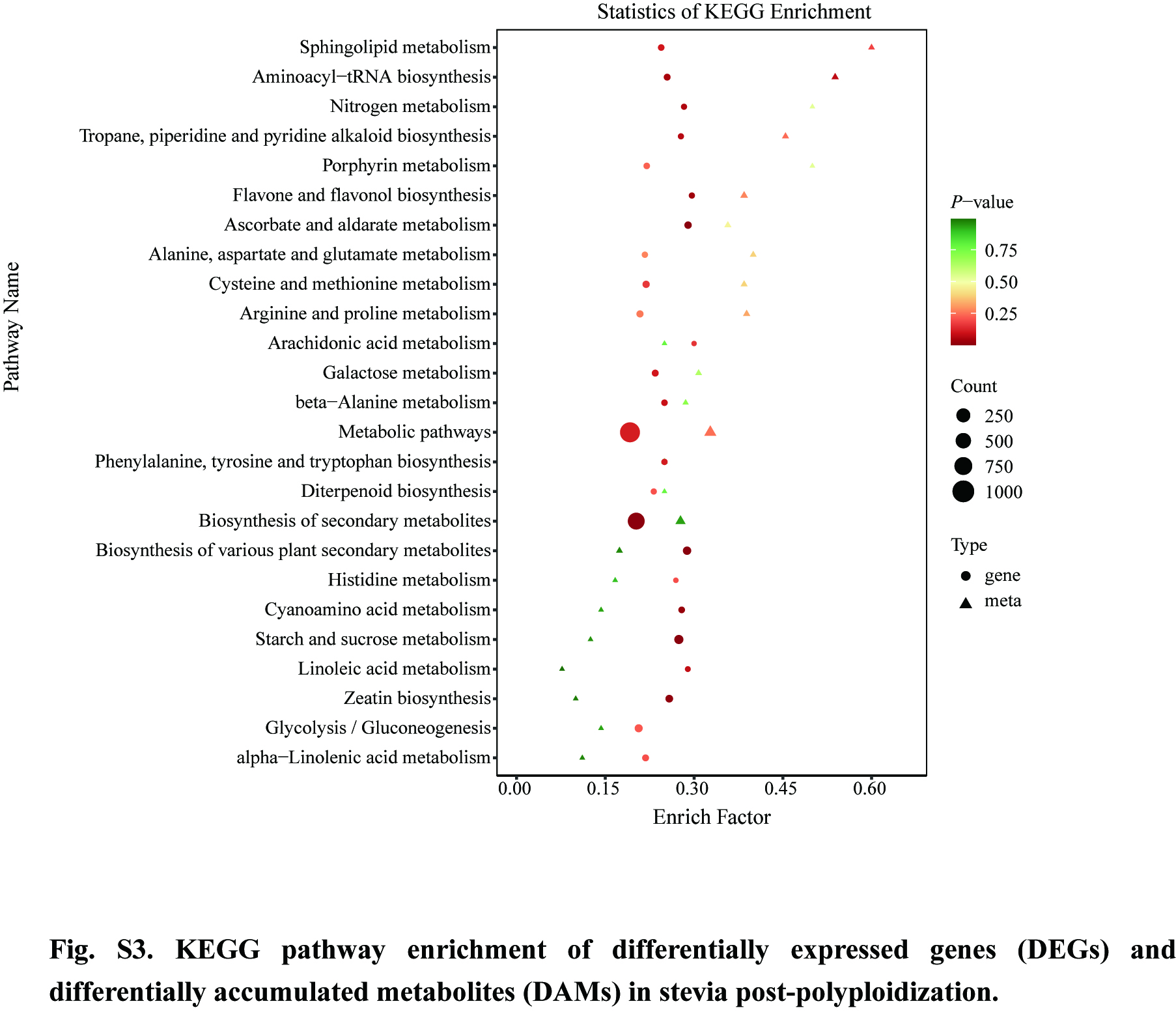

Supplement: Supplementary file 1 [file plants-13-02542-s001.zip › Fig. S3.jpg]

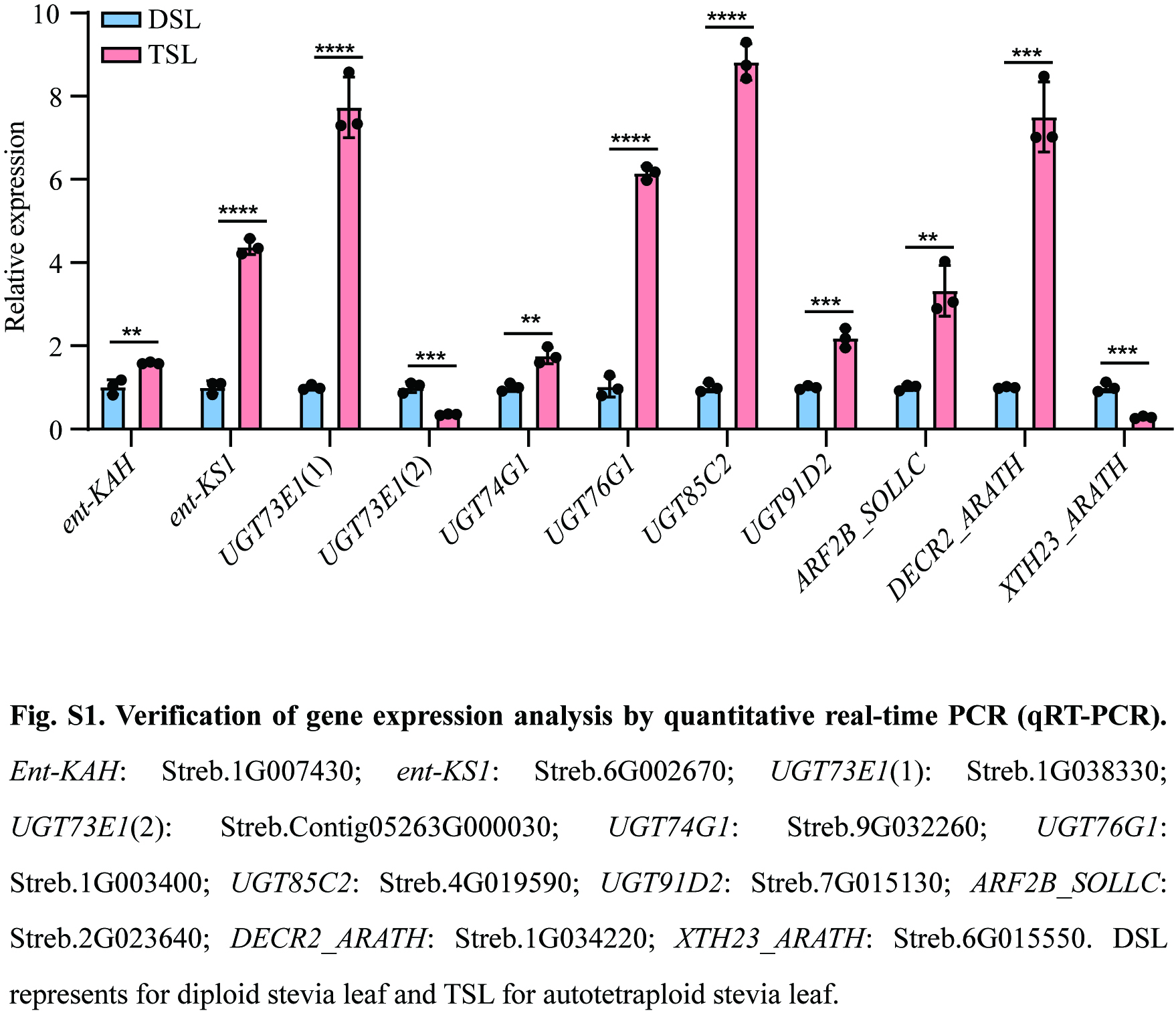

Supplement: Supplementary file 1 [file plants-13-02542-s001.zip › Fig.S1.jpg]

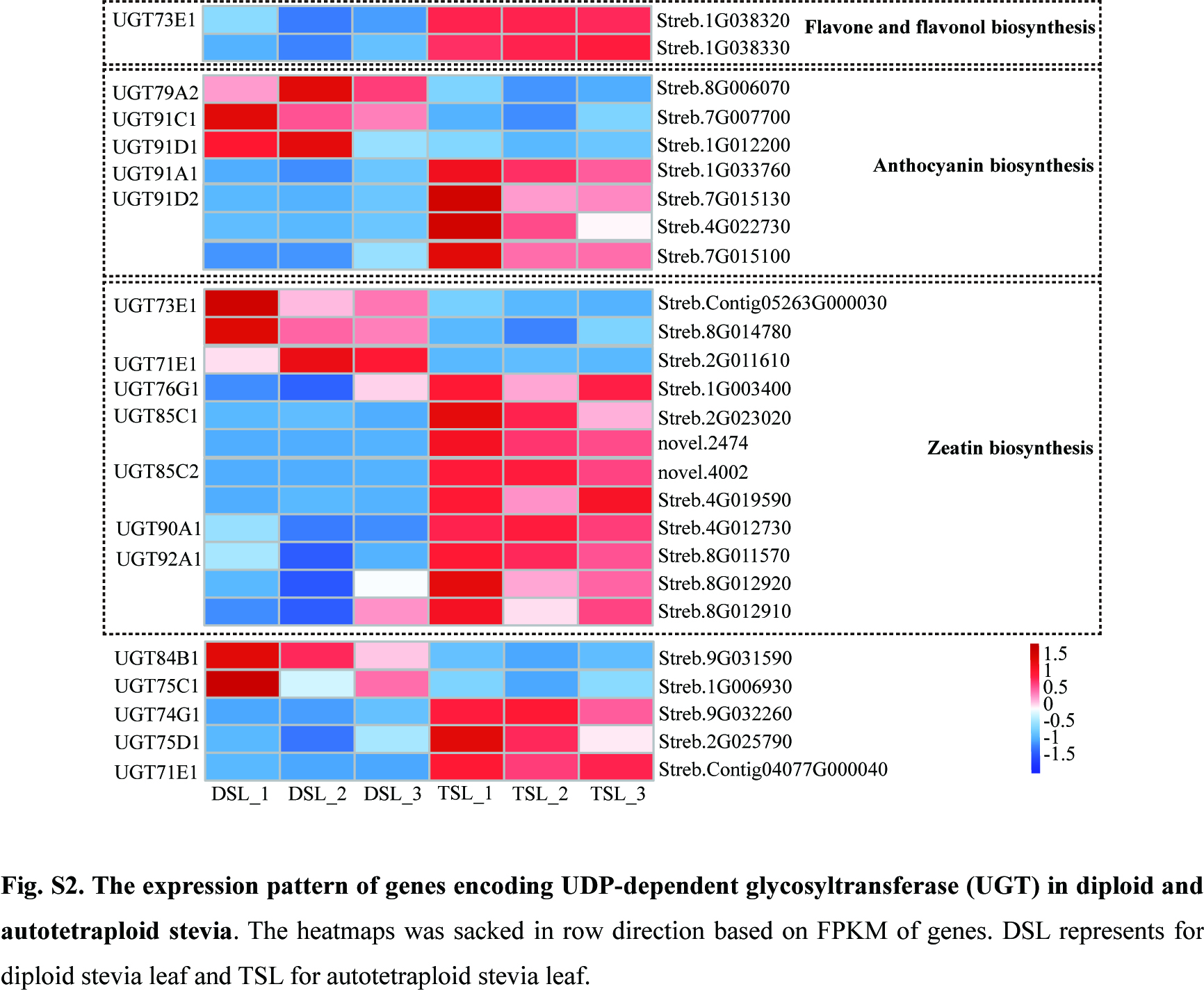

Supplement: Supplementary file 1 [file plants-13-02542-s001.zip › Fig.S2.jpg]
